# Novel Mouse Tauopathy Model for Repetitive Mild Traumatic Brain Injury: Evaluation of Long-Term Effects on Cognition and Biomarker Levels After Therapeutic Inhibition of Tau Phosphorylation

**DOI:** 10.3389/fneur.2019.00124

**Published:** 2019-03-11

**Authors:** Richard Rubenstein, Deep R. Sharma, Binggong Chang, Nassima Oumata, Morgane Cam, Lise Vaucelle, Mattias F. Lindberg, Allen Chiu, Thomas Wisniewski, Kevin K. W. Wang, Laurent Meijer

**Affiliations:** ^1^Laboratory of Neurodegenerative Diseases and CNS Biomarker Discovery, Departments of Neurology and Physiology/Pharmacology, SUNY Downstate Medical Center, Brooklyn, NY, United States; ^2^ManRos Therapeutics, Centre de Perharidy, Roscoff, France; ^3^Center for Cognitive Neurology and Departments of Neurology, Pathology and Psychiatry, New York University School of Medicine, New York, NY, United States; ^4^Program for Neurotrauma, Neuroproteomics and Biomarker Research, Departments of Emergency Medicine, Psychiatry and Neuroscience, University of Florida, Gainesville, FL, United States

**Keywords:** traumatic brain injury, repetitive mild closed head injury, TghTau/PS1 transgenic mice, total tau and phosphorylated tau, cognition, brain and blood-based biomarkers, roscovitine, lithium chloride

## Abstract

Traumatic brain injury (TBI) is a risk factor for a group of neurodegenerative diseases termed tauopathies, which includes Alzheimer's disease and chronic traumatic encephalopathy (CTE). Although TBI is stratified by impact severity as either mild (m), moderate or severe, mTBI is the most common and the most difficult to diagnose. Tauopathies are pathologically related by the accumulation of hyperphosphorylated tau (P-tau) and increased total tau (T-tau). Here we describe: (i) a novel human tau-expressing transgenic mouse model, TghTau/PS1, to study repetitive mild closed head injury (rmCHI), (ii) quantitative comparison of T-tau and P-tau from brain and plasma in TghTau/PS1 mice over a 12 month period following rmCHI (and sham), (iii) the usefulness of P-tau as an early- and late-stage blood-based biochemical biomarker for rmCHI, (iii) the influence of kinase-targeted therapeutic intervention on rmCHI-associated cognitive deficits using a combination of lithium chloride (LiCl) and R-roscovitine (ros), and (iv) correlation of behavioral and cognitive changes with concentrations of the brain and blood-based T-tau and P-tau. Compared to sham-treated mice, behavior changes and cognitive deficits of rmCHI-treated TghTau/PS1 mice correlated with increases in both cortex and plasma T-tau and P-tau levels over 12 months. In addition, T-tau, but more predominantly P-tau, levels were significantly reduced in the cortex and plasma by LiCl + ros approaching the biomarker levels in sham and drug-treated sham mice (the drugs had only modest effects on the T-tau and P-tau levels in sham mice) throughout the 12 month study period. Furthermore, although we also observed a reversal of the abnormal behavior and cognitive deficits in the drug-treated rmCHI mice (compared to the untreated rmCHI mice) throughout the time course, these drug-treated effects were most pronounced up until 10 and 12 months where the abnormal behavior and cognition deficits began to gradually increase. These studies describe: (a) a translational relevant animal model for TBI-linked tauopathies, and (b) utilization of T-tau and P-tau as rmCHI biomarkers in plasma to monitor novel therapeutic strategies and treatment regimens for these neurodegenerative diseases.

## Introduction

Traumatic brain injury (TBI) is generally defined as a closed head injury resulting from acceleration/deceleration forces and is separated into three categories: severe (sTBI), moderate (modTBI) and mild (mTBI). TBI is not a single independent event but can be considered as a disorder of broad-ranging consequences since the forces of head trauma vary in intensity and their effects on the brain involve functional, cellular and molecular changes. Many epidemiological studies provide evidence that sustaining a single or multiple TBIs are associated with increased risk for degenerative conditions that may result in dementia including Alzheimer's disease (AD). Both sTBI and modTBI are often associated with intracranial bleeding and diagnosis can be made by a clinical examination and neuroimaging (CT, MRI). On the other hand mTBI, also known as concussions, are generally considered head injuries that may or may not be associated with a short-term loss of consciousness of <30 min (1, 2). The transient and heterogeneous nature of mTBI symptoms makes it difficult to diagnose and, as a result, a large portion of these injuries go unrecognized. This is of major concern since 80–90% of all head injuries are cases of mTBI (1, 2). Among those who have had an mTBI, during the first year ~50% will continue to experience cognitive, neurological, and behavioral symptoms such as headache, difficulty concentrating, anxiety, and depression (3). After the first year, these numbers are typically reduced but not abolished. Furthermore, repetitive mTBI (rmTBI), usually associated with athletes participating in contact sports or military personnel, is a risk factor for a chronic progressive neurodegenerative disorder known as chronic traumatic encephalopathy (CTE). CTE is a progressive neurological disease that currently can only be diagnosed post-mortem. A post-mortem diagnosis depends mainly on the detection of abnormal hyperphosphorylated-tau (P-tau) neuropathology that is characterized by an irregular, focal, perivascular distribution largely localized to cortical sulci. Although P-tau is not unique to CTE, this distribution pattern helps distinguish it from the other P-tau linked chronic neurodegenerative diseases (CNDs), or tauopathies, which includes AD. Post-mortem identification of P-tau-containing neurofibrillary tangles (NFTs) are a key diagnostic marker used clinically to identify and distinguish the tauopathies (4).

Early diagnosis and prognosis of TBI has been a major area of interest. Since TBI is a heterogeneous disorder, treatment regimens can be optimized utilizing a precision medicine approach that would benefit from the identification and monitoring of biomarkers. Tau hyperphosphorylation appears to correlate with the neuropathological and neuropsychiatric deficits representative of neurotrauma-related neurodegeneration (5, 6). This is especially relevant not only in relation to the severity of TBI but also to the CNDs. Rubenstein et al. (7) reported increases of T-tau and P-tau in the CSF and serum of acute TBI patients. In the chronic TBI stage serum T-tau returned to basal levels while P-tau levels remained higher than control serum (7) suggesting that P-tau is indicative of axonal injury and is useful for assessing the progression of injury/recovery. The ability to target the inhibition or prevention of tau hyperphosphorylation would interfere with the neuropathological progression. Thus, it would be beneficial to use P-tau as both a biomarker for diagnosis, prognosis, and treatment monitoring as well as a therapeutic target. In addition to the identification of specific biochemical biomarkers, an animal model to study acute and chronic TBI which progresses into a CTE-like tauopathy would be useful not only to study the neuropathophysiology of the CNDs but also serve to establish a translational-relevant model for assessing targets and monitor effectiveness of therapeutic intervention during acute and chronic stages.

Numerous kinases can phosphorylate tau to different degrees. Glycogen synthase kinase 3β (GSK-3β) and cyclin-dependent kinase 5 (cdk5), both proline-directed serine/threonine kinases that have increased activity following TBI, are the two most potent kinases responsible for tau hyperphosphorylation (8–14). Kabadi et al. (15) reported that following TBI in mice, roscovitine (ros) not only reduced progressive neurodegeneration in the hippocampus and cortex, but also mitigated cortical neuroinflammation. In addition, ros also attenuated cognitive deficits and improved spatial learning and memory. Ros is a relatively selective cyclin-dependent kinase (cdk) inhibitor, which acts specifically on cdks 1, 2, and 5 as well as on cdks 7 and 9 (16, 17) and casein kinase 1 to a lesser extent (18). Lithium exerts neuroprotective effects by inhibiting GSK-3β (19). In various experimental TBI paradigms, lithium has been shown to not only reduce neuronal death, microglial activation, cyclooxygenase-2 induction, amyloid β, and hyperphosphorylated tau levels, but also to preserve blood-brain barrier (BBB) integrity and alleviate TBI-induced neurological deficits (20). We hypothesize that simultaneously inhibiting both cdk5 and GSK-3β can effectively reduce or prevent TBI-induced tau pathology.

Although blood-based biochemical markers have been successfully used in the diagnosis and monitoring of several diseases, they have had limited success in TBI. The plasma biomarkers, neurofilament-H, glial fibrillary acidic protein (GFAP), ubiquitin C-terminal hydrolase–L1 (UCHL1), neuron specific enolase, myelin basic protein, tau, and s100β, have been consistently demonstrated to be elevated during the acute phase of sTBI (21–27). The levels of these biomarkers in the blood reflect the extent of neuronal and glial damage and loss suggesting their usefulness for demonstrating injury severity and in predicting clinical outcomes especially in the acute phase of sTBI. However, some of these biomarkers are not exclusively CNS-specific which can compromise their value for a TBI diagnosis and prognosis. In human TBI patients, GFAP and UCH-L1 have been reported as early stage neurotrauma biomarkers for astrocytosis and neuronal cell damage, respectively (27). Increased UCH-L1 concentrations have been linked to injury severity and worse outcome after TBI while GFAP has been found to correlate with axonal injury, elevated intracranial pressure and mortality (28–31). The usefulness of these two biomarkers for diagnosing mTBI, however, is still unclear (32).

The microtubule-associated protein tau is abundant in axons (33) and functions to stabilize microtubules, influence axonal transport and regulates cytoskeleton organization. Tau-regulated normal cellular physiological functions become abnormal in response to head trauma (34). Hyperphosphorylation of tau as a result of TBI reduces the binding affinity of tau to microtubules causing microtubule destabilization and altered axonal transport leading to impaired neuronal function and neuropathophysiology (35). Animal models have shown a link between mTBI, increased P-tau depositions and neurobehavioral abnormalities (35). Previously studies with human plasma have suggested that the levels of P-tau and the P-tau:T-tau ratio might serve as useful biomarkers for acute and chronic mTBI (36).

In the present study we have measured the levels of T-tau and P-tau in plasma and cortical brain regions over a 12 month period following repetitive mild closed head injury (rmCHI) of a genetically-modified, tauopathy-prone transgenic mouse model termed TghTau/PS1 (37, 38). The TghTau/PS1 mouse line was previously developed and used as a model for AD to study immunotherapeutic targeting of tau pathology (38). This transgenic mouse line was generated by crossing TghTau mice (39) with a mouse model that expresses the PS1 M146L human mutation (40). The TghTau/PS1 mice are on a mouse tau knockout background, express full-length non-mutated human tau and have an earlier onset and more aggressive progression of tau pathology than the TghTau model. To our knowledge, TghTau/PS1 mice have not been used previously to study rmCHI. We correlated these biochemical data with the degree of cognitive deficits. Further, we determined whether a drug treatment regimen targeting protein kinases responsible for tau phosphorylation can reverse the detrimental effects of rmCHI. Quantitation of biomarker levels in the plasma and cortex of TghTau/PS1 mice has been carried out using the a-EIMAF and EIMAF laser-based technologies (7), respectively. Using a-EIMAF and EIMAF, we were able to detect concentrations of T-tau and P-tau throughout the 12 month study period. This not only stresses the usefulness of the technology for blood-based and brain biomarker levels during the early and late stages of rmTBI but also continues to implicate the usefulness of P-tau as a blood-derived biomarker not only for TBI (36) but also preclinically and clinically for tauopathies such as AD, frontotemporal lobar degeneration and CTE.

## Materials and Methods

### Animals

TghTau/PS1 mice were bred in-house and maintained under standard laboratory conditions (23 ± 1°C, 50 ± 5% humidity, and 12-h light/dark cycle) with free access to food and water throughout the study.

### Injury Protocol and Drug Treatments

**Figure 1:**
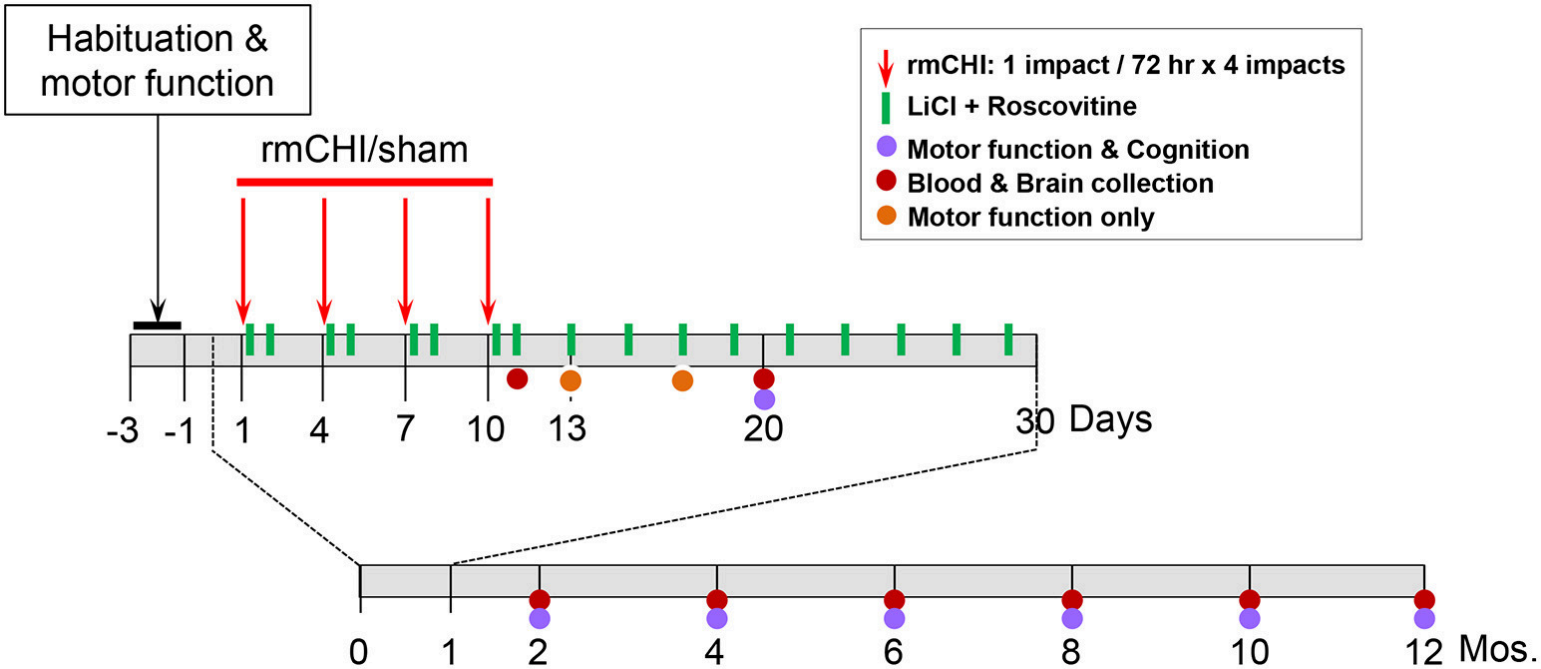
Schematic representation of the overall study design.

Our rmCHI study design is described schematically (Figure 1). We established the optimal parameters for rmCHI of TghTau/PS1 mice guided by previous reports (41, 42) and confirmed by empirically testing conditions prior to initiating the studies described in this manuscript. Our requirements for establishing the appropriate set of rmCHI conditions included: (i) a short period (<30 s) of post-impact apnea (recognized as an animal analog to human loss of consciousness (43), (ii) a short period (<6 min) of righting reflex (the time it takes for the mouse to right itself onto all four paws), (iii) no signs of skull fracture at the time of euthanasia, and (iv) no history of major hemorrhage(s). Based on our preliminary testing and requirements, we determined that the optimum parameters for rmCHI of TghTau/PS1 mice consisted of each impact having a: (i) velocity = 5.0 m/s, (ii) compression = 1 mm, and (iii) dwell = 200 msec. Each mouse was given an interconcussion interval of 72 h between each of 4 impacts (one impact per day on days 1, 4, 7, 10).

All studies were initiated on 3–3.5 month old TghTau/PS1 mice. For each time point of our study design (1 day, 10 days, 2, 4, 6, 8, 10, and 12 mos.) 40-44 male and female TghTau/PS1 mice (randomly selected with similar gender distribution) were subjected to rmCHI and equal numbers of randomized, gender-distributed mice were sham-treated. All mice were anesthetized with 2.5% isoflurane in a 1:1 oxygen/air mixture. After reaching a deep level of anesthesia, the skull areas of the mice were shaved and mounted on a stereotactic frame in a prone position with the head placed under and touching a 4 mm blunt metal impactor tip that was positioned midway relative to the sagittal suture. For mice that were receiving a closed head injury, the metal impactor tip was retracted, adjusted to a 1 mm downward position and released electronically using the MyneuroLab (St. Louis, MO) controller thus subjecting the animal to a single closed head neurotrauma caused by the impactor tip having a downward velocity of 5 m/s and a 1 mm deep impact to the skull which lasted 200 ms before the impactor metal tip automatically retracted back to its original position. At the end of the procedure, each animal was removed from the stereotaxic table, allowed to recover on a heating pad, and, upon becoming ambulatory, was returned to its home cage. Sham-treated animals underwent the same procedures, including exposure to anesthesia for the same length of time, as the rmCHI animals, but were not subjected to the impactor tip.

Half of the mice from the rmCHI and sham groups were randomly selected (while still maintaining the gender distributions) for drug administration or no drug treatments. Drug-treated rmCHI and sham-injured mice received a non-toxic combination of lithium chloride (LiCl) (150 mg/kg) and (R)-roscovitine (ros) (100 mg/kg) intraperitoneally (ip) at 3 and 24 h following each of the 4 impacts. Thereafter, the mice received the same drug combination once every 48 h until day 30 from the initial impact. No additional drug treatments were administered for the remainder of the 12 month study. The drug doses administered were based on both previous reports (16, 20, 44–46) and our objective exploratory studies which showed no toxicity in TghTau/PS1 mice at any of the doses we tested both individually or in combination up to the maximum of 300 mg/kg. The doses for our studies (150 mg/kg LiCl and 100 mg/kg ros) were selected based on the lowest doses that maximally inhibited P-tau at 24 and 48 h. following our 4-impact rmCHI and drug administration protocols described above. No deaths occurred as a result of the neurotrauma impact protocol or drug-associated toxicity throughout the 12 month study design.

### Motor Function, Behavior, and Cognition

Motor coordination with and without behavioral and cognitive analyses were performed on mice over the 12 month time course as indicated on the study design scheme (Figure 1). At the completion of cognitive testing at each time point, randomized groups of mice were either euthanized for blood and brain removal or returned to their cages within the animal facility for future analysis.

To assess motor function, an accelerating rotarod (Panlab, Harvard apparatus) was used for quantitative analysis of motor coordination and learning. The mice were placed on the rod that rotated at 4 rpm at 0 min and gradually accelerated to 40 rpm after 5 min of run time. Mice were oriented perpendicular to the long axis of the rod, such that the mice had to make forward walking movements to avoid falling. Mice received three training trials with a 10 min inter-trial interval followed by three “test” trials on which latency time to fall from the rod was measured and then the average latency time was used. Behavioral and cognitive analysis was performed once the rotarod confirmed that the rmCHI and sham mice had similar motor function.

Cognitive testing consisted of active place avoidance (APA) and conflict active avoidance (CAA) (47, 48) where an animal's spatial learning and memory is tested by its ability to avoid a shock zone within a rotating circular arena. The successful utilization of APA and CAA cognition testing in rodents have been published previously (49–53). APA represents initial learning and memory by testing if rmCHI alters the ability of the mice to actively avoid the shock zone following habituation of its location. APA requires the segregation of stationary distal visual cues and rotating proximal olfactory cues. CAA reflects learning, memory and reasoning when the animal is confronted with a conflicting environment. Functional testing was performed by an individual blinded to the treatment groups. The apparatus used for the APA and CAA behavioral tasks has been described previously (51, 52). Briefly, an apparatus arena was placed in a room with prominent visual landmarks on the walls. The arena was a rotating 33-cm diameter metal grid and a 33-cm high transparent wall that allowed the mice to view the room. The position of a mouse was followed by computer tracking using a camera mounted above the arena and an infrared light-emitting diode. Prior to APA analysis, each mouse was first given a 10 min pre-training session (open field) in which they are placed in the rotating arena. The pre-training acclimates the mouse to the environment and reduces anxiety. The mouse was then given four 10 min testing trials, with a 60 min inter-trial interval, in which the computer designated a 60°segment of the rotating arena as the shock-zone area. Upon entry into the shock zone, a mouse received a 0.2 mA shock of 60 Hz for 500 msec. through the grid floor. The mouse continued to receive shocks every 1.5 s until it exited the shock zone. The parameters monitored as indicators of learning and memory included: (i) the number of shock zone entrances during each trial, (ii) time before the first entrance into the shock zone at the beginning of each trial and (iii) tracking mouse movement as well as distance traveled within the arena using track analysis software (BioSignal Group, Brooklyn, NY). CAA was initiated 1 day following APA and also consisted of four 10 min trials. In CAA the shock zone was shifted 180° from its APA location. CAA assessed an animal avoidance memory when it conflicted with the learned avoidance memory from APA on the previous day. An animal's anxiety was monitored by measuring both its distance traveled and continuous location within the arena. The time before the first entrance into the shock zone during each CAA trial and the total number of entrances into the shock zone measured how well the mouse learned and remembered the new shock zone location. During all APA and CAA testing, mice were returned to their home cages outside of the testing room between multiple testing periods and to the main animal facility at the completion of analysis each day. Both the apparatus and the dedicated testing room were unchanged throughout the entire 12 month study.

### Sample Preparation and Quantitative Biomarker Analysis

Ten percent mouse cortical brain homogenates were prepared on ice in Tris-buffered saline (TBS) (10 mM Tris-HCl, 140 mM NaCl), pH 7.4, containing 10 mM sodium fluoride, 2 mM EGTA, 1 mM sodium vanadate and 100x protease inhibitor cocktail (Fisher Scientific). To the homogenates was added 0.04 volumes of 5 M NaCl and beta-mercaptoethanol to 5% (v/v) final. The samples were boiled at 100°C for 10 min and incubated on ice for 30 min. Samples were microfuged at 4°C for 15 min at 10,000 x g, followed by collection and frozen storage of supernatants. Protein concentrations of supernatants were determined using the Pierce Bradford assay (ThermoFisher Scientific). T-tau and P-tau were detected and quantitated on diluted supernatants using the EIMAF (Enhanced Immunoassay using Multi-Arrayed Fiberoptics) technology (7).

Blood was collected into heparinized tubes and centrifuged. The plasma fraction was saved and quick-frozen. Biochemical detection and quantitation of T-tau and P-tau were performed using EIMAF conjugated to rolling circle amplification (a-EIMAF) (7).

### Cell Culture and Immunoblotting

**Figure 2:**
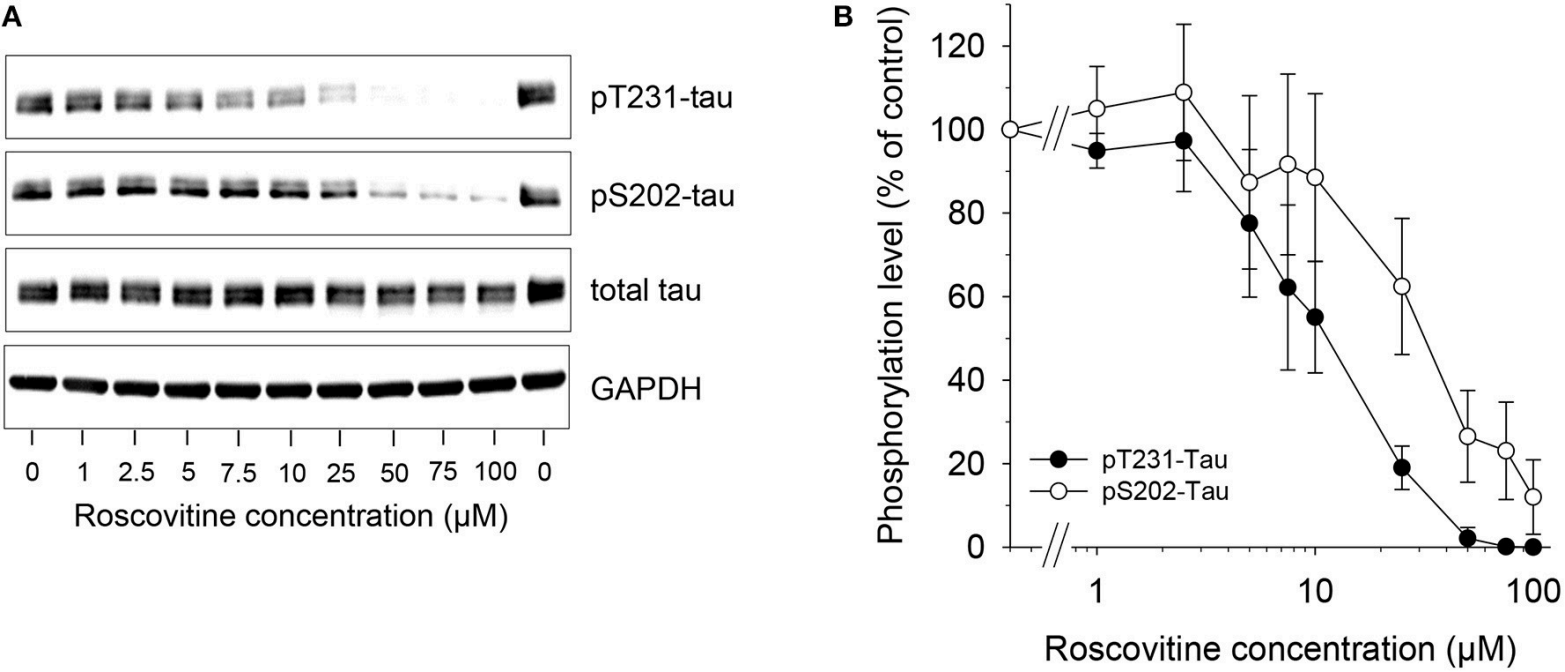
Dose-dependent down-regulation of tau phosphorylation is induced by roscovitine. SH-SY5Y-(P301L) cells were exposed for 6 h to a range of roscovitine concentrations. Cells were harvested and proteins were resolved by SDS-PAGE followed by western blotting **(Left)** with antibodies to pThr231 (Mab RZ3), pSer202 (Mab CP13), and T-tau (Mab DA31). α-GAPDH was used as a loading control. Western blotted protein bands were quantified (mean ± SE of three independent experiments) **(Right)**.

**Figure 3:**
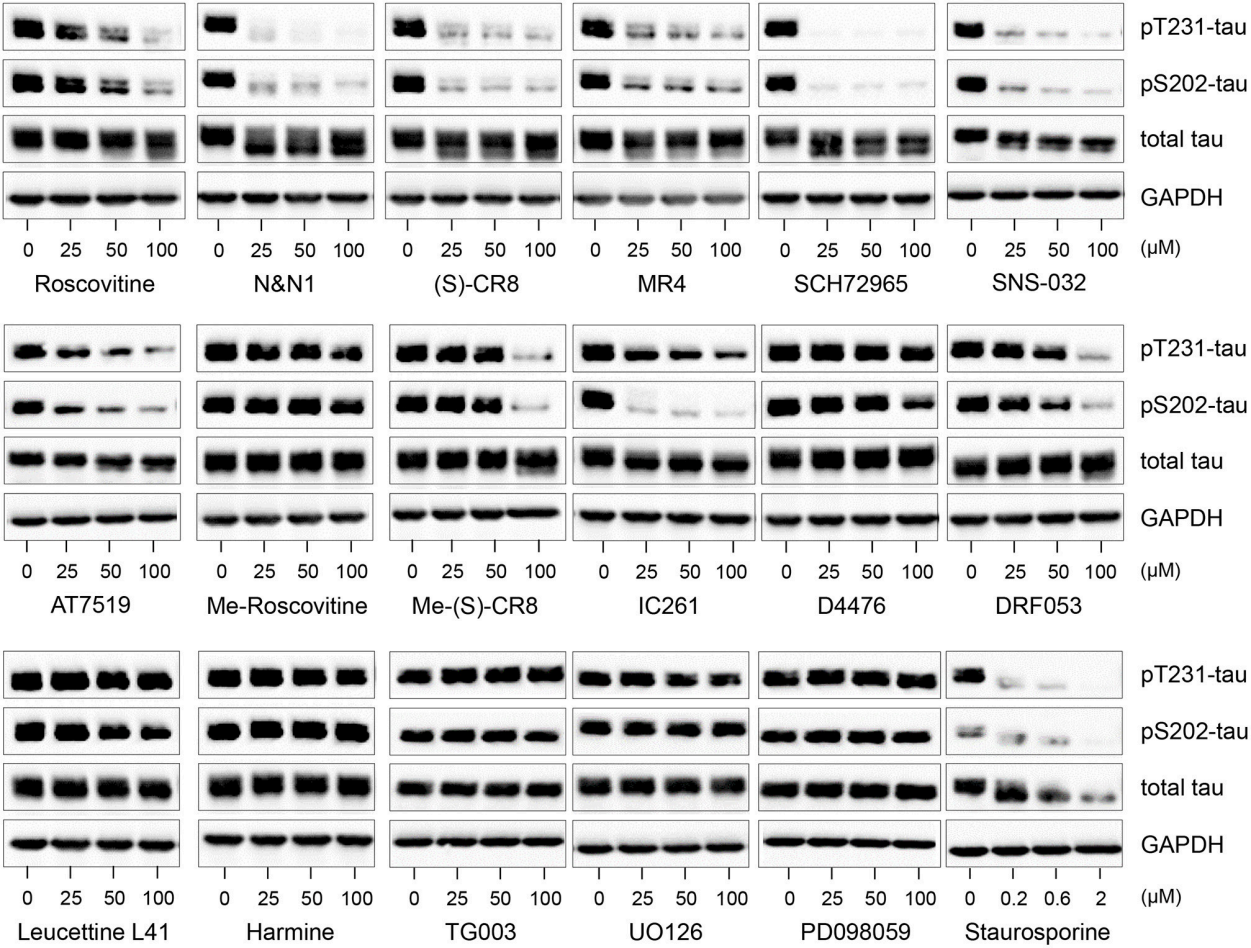
Decreased tau phosphorylation by roscovitine is due to its inhibition of cyclin-dependent kinases (CDKs) and not other kinase targets. SH-SY5Y-(P301L) cells were exposed for 6 h to pharmacological inhibitors of CDKs (roscovitine, N&N1, (S)-CR8, MR4, SCH72965, SNS-032, AT7519), to kinase inactive derivatives of roscovitine (N6-methyl-roscovitine) or CR8 (N6-methyl-CR8), CK1 (IC261, D4476, DRF053), DYRKs/CLKs (Leucettine L41, harmine, TG003), MEK1 (UO126, PD098059), to a general kinase inhibitor (staurosporine). All drugs were tested at 25, 50, and 100 μM, except staurosporine (0.2, 0.6, and 2 μM). Cells were harvested and proteins were resolved by SDS-PAGE followed by western blotting with antibodies to pThr231 (Mab RZ3), pSer202 (Mab CP13), and T-tau (DA31). α-GAPDH was used as a loading control.

**Figure 4:**
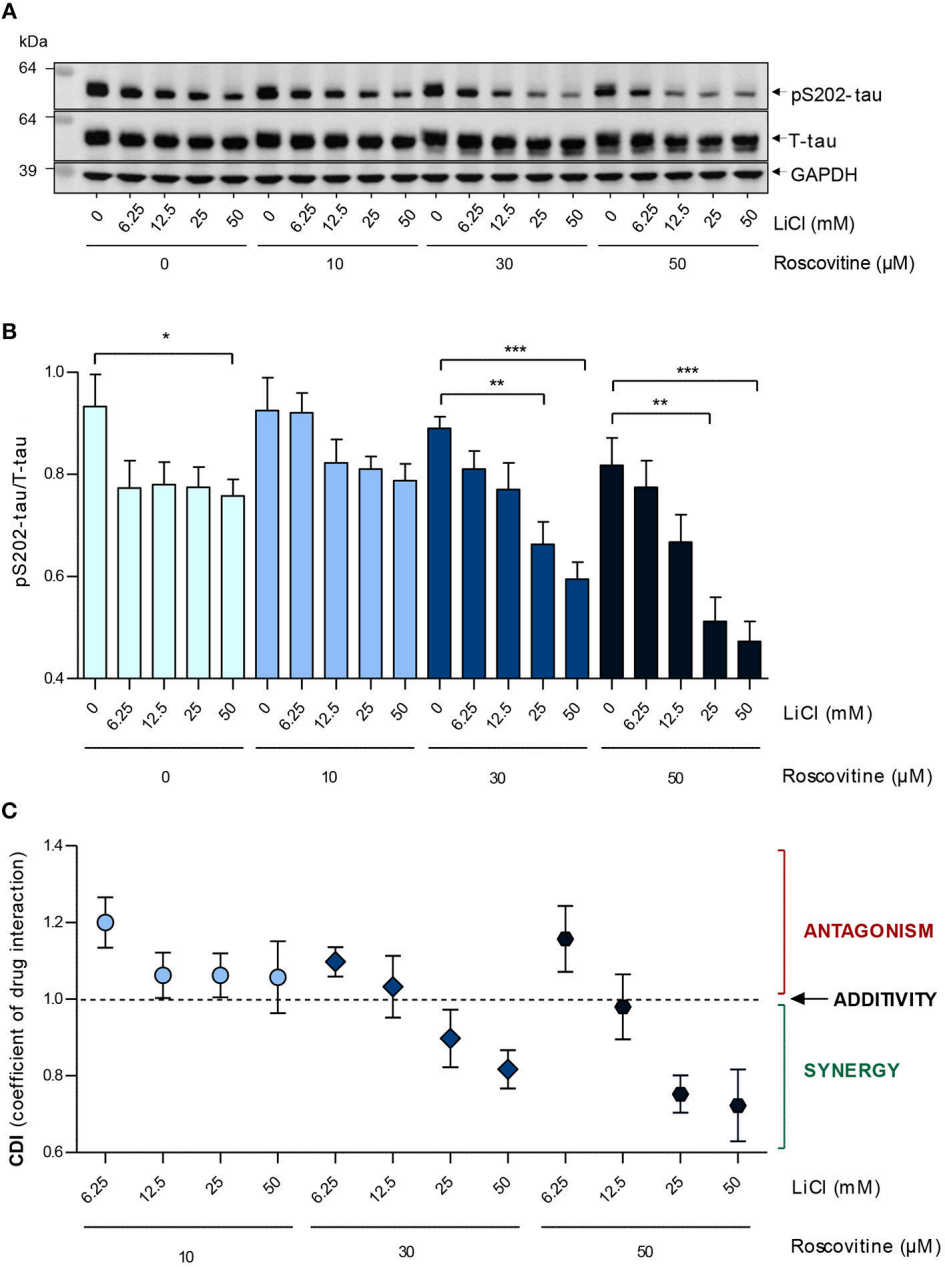
Roscovitine induces a dose-dependent down-regulation of S202-Tau phosphorylation in SH-SY5Y-(P301L) cells and synergizes with LiCl. SH-SY5Y-(P301L) cells were exposed for 6 h to a range of roscovitine and/or LiCl concentrations. Cells were harvested and proteins were resolved by SDS-PAGE followed by Western blotting **(A)** with pS202-tau (Mab CP13) and T-tau (Mab DA9). α-GAPDH was used as a loading control. Western blotted protein bands were quantified and normalized to GAPDH, and are presented as pS202-tau/T-tau ratios [**(B)**, left panel, mean ± SEM of four independent experiments]. Coefficients of drug interactions (CDI) show synergistic effects at concentrations ≥25mM LiCl and 30 μM roscovitine **(C)**. The *p*-values are depicted with an asterisk (^*^p < 0.05, ^**^p < 0.01, ^***^p < 0.001; two-tailed Student's *t*-test).

SH-SY5Y-P301L cells (gift from Prof. Fred Van Leuven, Department of Human Genetics, KU Leuven, Belgium) were cultured Dulbecco's Modified Eagle Medium (DMEM):Nutrient Mixture F-12 (DMEM/F-12, Gibco, c/o Invitrogen, Saint Aubin, France) containing 1% Penicillin-Streptomycin mixture (Gibco) and 10% fetal bovine serum (FBS, Gibco) in a humidified, 5% CO_2_ incubator at 37°C. Cells were split routinely once a week. Cells were seeded at a density of 1.5 x 10^6^ cells/well in P60 well plates with 4 ml medium/well and exposed for 6 h to a range of ros concentrations (Figures 2–4) or to a few concentrations of various kinase inhibitors with different selectivities (Figure 3). At the end of the incubation cells were harvested for Western blotting. Cells were scraped in cold phosphate-buffered saline, centrifuged at 10,000 x g for 5 min at 4°C, and pellets were snap-frozen in liquid nitrogen. Experiments were repeated three or four times.

For Western blot analysis, cell pellets were lysed in homogenization buffer (25 mM MOPS, 15 mM EGTA, 15 mM MgCl_2_, 60 mM β-glycerophosphate, 15 mM *p*-nitrophenylphosphate, 2 mM dithiothreitol (DTT), 1 mM Na_3_VO_4_, 1 mM NaF, 1 mM disodium phenylphosphate, 1x protease inhibitor cocktail, 0.2% Nonidet P-40 substitute). Protein extracts were mixed (1:1 v/v) with sample buffer (2 × NuPAGE LDS sample buffer, 200 mM DTT) and 20 μg of total protein was loaded on NuPAGE 4–12% Bis-Tris protein gels. Electrophoresis was carried out at 70 V in MOPS buffer for 4 h. Rapid blot transfers were performed at 2.5A/25V for 7 min. Blotting membranes were blocked in milk (5% Regilait in TBS with 0.1% Tween (TBST) for 1 h. The membranes were incubated overnight at 4°C with anti-tau monoclonal antibodies (Mab) RZ3 (pThr231) or Mab CP13 (pSer202), T-tau (Mab DA9) and anti-GAPDH antibody (1:30,000 dilution; Bio-Rad, Marnes-la-Coquette, France) followed by incubation for 1 h at room temperature with goat anti-rabbit or goat anti-mouse antibodies (Bio-Rad). Chemiluminescent detection was achieved with homemade ECL-Tris buffer (100 mM Tris pH 8.5, 0.009% H_2_O_2_, 0.225 mM p-coumaric acid, 1.25 mM luminol) and Fusion F × 7 camera software. P-tau and T-tau signals were first normalized to GAPDH from the same membranes, and then divided one by another to obtain the level of pS202 or pT231 relative to T-tau. Values are presented as mean±SEM of three or four independent experiments.

### Mass Spectroscopy

#### Interactomics Study of Roscovitine Using Competition Affinity Chromatography

The method is based on an affinity matrix comprising a set of well-characterized broad-spectrum kinase inhibitors (KinAffinity® developed by Kinaxo/Evotec) to enrich the subproteome of endogenously expressed kinases of cells or tissues (54). Kinase inhibitors are screened against these matrix-bound proteins to reveal the inhibitor's quantitative cellular target profile. First, a lysate of mouse brain was applied to the KinAffinity® matrix comprising a mixture of various immobilized broad-band kinase inhibitors, adjusted to different concentrations. These quantitative binding experiments determines the concentration of the immobilized compounds required to obtain 50% binding of each target protein to the matrix (BC_50_). Second, a lysate of mouse brain was applied to the KinAffinity® matrix with the highest loading density of kinase inhibitors in the presence of increasing concentrations of free ros. These quantitative competition experiments determine the free compound concentration required for 50% target protein to remain bound to the matrix (CC_50_). The final K_d, free_ values for the free compound were calculated for each target protein using the Cheng-Prusoff equation (55).

#### *In vitro* Association Experiments

C57Bl/6J mouse brain tissue (2.5 gm) was frozen in liquid nitrogen and prepared as a fine powder by grinding with a mortar and pestle. The fine powder was resuspended in 15 ml lysis buffer containing 20 mM HEPES pH 7.5, 150 mM NaCl, 0.25% Triton X-100, 1 mM EDTA, 1 mM EGTA, 1 mM DTT plus additives (10 μg/ml aprotinin, 10 μg/ml leupeptin, 1 mM PMSF, 1 mM Na_3_VO_4_, 10 mM NaF). The suspension was homogenized on ice for 3 x 30 s using a Ultra-Turrax T8 disperser equipped with a S10N-5G dispersing element (IKA). *In vitro* association experiments were performed as previously described (56) with the exception that KinAffinity^TM^ beads (Kinaxo Biotechnologies), representing a set of different broad-spectrum kinase inhibitors immobilized on Sepharose beads, were applied for affinity chromatography and for competition experiments. Tissue extracts were treated with different concentrations of ros (0, 1, 10, 100 nM, 1, 10, 100 μM) for 30 min prior to addition of inhibitor beads and incubation for an additional 2.5 h at 4°C. Alternatively, free inhibitor and KinAffinity^TM^ beads were added simultaneously to the tissue extract.

#### Mass Spectrometry Sample Preparation

Proteins enriched with the KinAffinity^TM^ beads were digested on the beads using an in-solution procedure. Briefly, per condition, 100 μl digestion buffer (8 M urea, 50 mM Tris pH 8.2, 10 mM sodium pyrophosphate, 5 mM EDTA, 5 mM EGTA, 10 mM NaF, 10 mM β-glycerophosphate, 10 mM Na_3_VO_4_, phosphatase inhibitor cocktail 2 and 3 (Sigma, 1:100 (v/v)) and Complete Protease Inhibitor Cocktail Tablets (Roche) were added. Samples were then reduced with 1 mM DTT for 30 min at room temperature (RT) and then alkylated with 5.5 mM chloroacetamide for 45 min at RT. Proteins were initially cleaved with lysyl endopeptidase (Wako) (0.3 μg per sample) for 4 h and diluted 5-times with 20 mM Tris/HCl (pH 8.2) prior to overnight proteolytic cleavage with trypsin (Promega) (0.5 μg/sample). The peptide mixtures were acidified by the addition of trifluoroacetic acid to a final concentration of 0.4% and subsequently desalted via C_18_ Sep-Pak columns (Waters). Peptides were eluted with 50% acetonitrile, 0.5% acetic acid, frozen in liquid nitrogen and lyophilized. To enable subsequent quantitative MS-analysis, peptides were labeled by means of stable isotope dimethyl labeling as described (57). Briefly, dried peptides of one sample were reconstituted in 100 μl of 100 mM triethylammonium bicarbonate (pH 5–8.5) and substituted with the respective formaldehyde- and sodium cyanoborohydride solutions for the labeling reactions to result in light (Δ 28 m/z), medium (Δ 32 m/z), or heavy (Δ 36 m/z) peptide entities. After quenching the labeling reactions, the light-, medium- and heavy-peptide entities belonging to one KinAffinity^TM^ experiment were combined, frozen in liquid nitrogen and lyophilized. To reduce sample complexity, each sample was separated into three fractions prior to LC-MS analysis. Therefore, samples were reconstituted in 100 μl of SCX buffer A (7 mM KH_2_PO_4_, 30% acetonitrile, pH 2.65) and loaded onto in-house prepared cation-StageTips (3 M, Empore™ Cation-SR). Fraction 1 represents the flow-through combined with an elution at 15% SCX buffer B (SCX buffer A containing 350 mM KCl), fraction 2 and 3 contain peptides eluted with 45 and 100% SCX buffer B, respectively. Peptides of each fraction were then desalted and enriched using in-house made C_18_ STAGE Tip columns (58). Eluted peptides were concentrated to a final volume of 2 μl in a vacuum concentrator 5,301 (Eppendorf) and substituted with 10 μl of 0.5% acetic acid before MS analysis.

#### Mass Spectrometry Analysis

LC-MS/MS analysis was performed on an LTQ-Orbitrap Velos mass spectrometer (ThermoFisher Scientific). A sample was loaded by an Easy n-LC II nanoflow system (ThermoFisher Scientific) on a 15 cm fused silica emitter (New Objective) packed in-house with reversed phase material (Reprusil-Pur C18-AQ, 3 μm) at a flow rate of 500 nl/min. The bound peptides were eluted by 140 min runs comprised of a segmented gradient from 10 to 30% of solvent B (80% acetonitrile, 0.5% acetic acid) over 96 min, 30 to 50% of solvent B over 12 min and 50 to 60% of solvent B over 7 min at a flow rate of 200 nl/min. Peptides were sprayed directly into the mass spectrometer using a nanoelectrospray ion source (ProxeonBiosystems). The mass spectrometer was operated in the positive ion mode with application of a data dependent mode to automatically switch between MS and MS/MS acquisition and a dynamic exclusion for the subsequent 90 s of ions once selected for fragmentation. To improve mass accuracy in the MS mode, the lock-mass option was enabled as described (59). Full scans were acquired in the orbitrap at a resolution R = 60,000 and a target value of 1,000,000 ions. The 15 most intense ions detected in the MS scan were selected for collision-induced dissociation in the LTQ mass spectrometer at a target value of 5,000 ion counts. General used mass spectrometric settings were: spray voltage, 2.2 kV; no sheath and auxiliary gas flow; heated capillary temperature, 220°C; normalized collision energy, 35% and an activation q = 0.25.

#### Data Processing

MS raw files were collectively processed with the MaxQuant software suite version 1.2.0.32 (60). Peak lists were searched against the mouse swissprot database version 2011_07 using the Andromeda search engine (61). Trypsin was selected as proteolytic enzyme and we required a minimal peptide length of 6 amino acids and maximal 2 missed cleavage sites were allowed. Carbamidomethylation of cysteine residues was set as fixed modification while oxidation of methionine and protein N-acetylation were allowed as variable modifications. For peptide quantification the “match between runs” function was enabled with a 2 min time window.

### Statistical Analyses

Statistical analyses of behavior and biochemical studies were performed using GraphPad Prism. For analysis of biochemical studies, two-way ANOVA with Tukey *post-hoc* for multiple comparisons was used to determine whether significant differences existed in biomarker concentrations between rmCHI and sham-treated mice at the different time points with and without drug treatment. Similarly, two-way ANOVA with Tukey *post-hoc* for multiple comparisons was also used for data analysis from behavioral studies. In all cases *p* < 0.05 was considered to be significant. All data are presented as the mean ± SEM.

For cell cultures and immunoblotting data the two-tailed unpaired *t*-test (Graphpad Prism) was used. Coefficients of drug interaction (CDI) were calculated as follows: CDI = AB/(A × B). According to the ratio pS202 tau/T-tau of each group, AB is the ratio of the combination groups to control group; A or B is the ratio of the single agent group to control group. Thus, CDI values <1, = 1, or >1 indicate that the drugs are synergistic, additive or antagonistic, respectively.

## Results

### Mass Spectroscopy

Lithium has been shown to directly and specifically inhibit GSK-3α and GSK-3β. LiCl treatment results in significantly decreased tau phosphorylation at putative GSK-3-directed sites, including Ser-202 and Ser-396/404.

However, although ros is a selective inhibitor of cyclin-dependent kinases (CDKs) (17) and casein kinases 1 (CK1s) (18), the specific targets of ros in the brain are less clear. CDKs and CK1s are families of protein kinases that have a spectrum of activities in regulating many cellular activities. Cdk5 has been proposed to be partially responsible for tau hyperphosphorylation. Based on our data showing that ros contributes to P-tau inhibition, we investigated the target specificity of ros in mouse brain using mass spectroscopy. Stable isotope chemical dimethyl labeled mouse brain proteins were loaded on KinAffinity^TM^ matrix. A detailed analysis of mouse brain kinases was performed (Table S1). A total of 150 distinct protein kinases and 7 lipid kinases were identified by high-end quantitative mass spectrometry following affinity chromatography purification (Table S1). Thirty proteins identified as ros targets were identified (Table 1). Of these, 23 protein kinases, 3 lipid kinases and 4 associated proteins were identified as the ros targets (Kd value < 40 μM) (Table 1). This data demonstrated that ros has multiple identified targets expressed in the brain. Although cdk5 appeared to be the main target in TBI, it possible that ros adds beneficial effects by inhibiting other CDKs as well as CK1s.

**Table 1:** Mouse brain Roscovitine-interacting proteins, either direct (like CDKs) or indirect (like cyclins).

**Roscovitine-interacting proteins**^a^			
**Uniprot ID**	**Protein**	**Synonym**	**K**_**d, free**_ **[μM]**
Q8BTW9	p21-activated kinase 4	PAK4	0.034
Q9QWV9	Cyclin-T1	CCNT1	0.046
Q99J95	Cyclin-dependent kinase 9	CDK9	0.090
P61809	Cyclin-dependent kinase 5 activator 1	CDK5R	0.108
Q9DC28	Casein kinase I isoform delta	CSNK1D	0.501
Q9JMK2	Casein kinase I isoform epsilon	CSNK1E	0.602
Q8BTH8	Casein kinase I isoform gamma-1	CSNK1G1	0.882
Q61214	Dual specificity tyrosine-phosphorylation-regulated kinase 1A	DYRK1A	1.000
Q9ESJ1	CDK5 and ABL1 enzyme substrate 1	CABLES	1.475
Q8C015	p21-activated kinase 5	PAK5	1.532
P61963	DDB1- and CUL4-associated factor 7	DCAF1	1.713
Q8C4X2	Casein kinase I isoform gamma-3	CSNK1G3	3.987
Q8BK63	Casein kinase I isoform alpha	CSNK1A1	7.368
P49615	Cyclin-dependent kinase 5	CDK5	7.666
O70172	1-phosphatidylinositol-5-phosphate 4-kinase 2-alpha	PIP4K2A	8.321
Q9WUT3	90 kDa ribosomal protein S6 kinase 2	p90RSK2	8.545
Q9Z2B9	90 kDa ribosomal protein S6 kinase 4	MSK2	9.596
Q04735	Cyclin-dependent kinase 16	CDK16	11.802
Q80XI4	1-phosphatidylinositol-5-phosphate 4-kinase 2-beta	PIP4K2B	12.399
P18653	90 kDa ribosomal protein S6 kinase 1	p90RSK1	14.677
Q04899	Cyclin-dependent kinase 18	CDK18	17.197
P18654	90 kDa ribosomal protein S6 kinase 3	p90RSK3	18.942
P34152	Focal adhesion kinase 1	FAK	19.798
P08414	Calcium/calmodulin-dependent protein kinase type IV	CAMK4	23.744
Q91XU3	Phosphatidylinositol-5-phosphate 4-kinase type II gamma	PIP4K2C	24.851
Q9QVP9	Proline-rich tyrosine kinase 2	PYK2	27.778
63085	Extracellular signal-regulated kinase 2	ERK2	33.945
Q63844	Extracellular signal-regulated kinase 1	ERK1	37.485
Q8K0D0	Cyclin-dependent kinase 17	CDK17	40.611
O35495	Cyclin-dependent kinase 14	CDK14	40.758

a *Shown are only kinase and non-kinase target proteins with a K_d, free_ value < 40 μM*.

### *In vitro* Studies

Tau phosphorylation in SH-SY5Y-(P301L) cells is inhibited by ros. A SH-SY5Y neuroblastoma cell line overexpressing P301L tau was used to investigate the effects of ros on the phosphorylation of tau at two specific sites. SH-SY5Y-(P301L) cells (1.5x10^6^ cells/well) were exposed for 6 h to a range of concentrations of ros. Cells were harvested, lysed and proteins were resolved by SDS-PAGE followed by Western blotting using anti-tau antibodies to pThr231 (Mab RZ3), pSer202 (Mab CP13), T-tau (Mab DA31), and GAPDH. Results demonstrated a dose-dependent inhibition of Thr231 and Ser202 phosphorylation (Figure 2, left panel). Phosphorylation of Thr231 appeared to be marginally more sensitive to ros inhibition than phosphorylation of Ser202 (Figure 2, right panel).

Ros reduces tau phosphorylation at Thr231 and Ser202 through inhibition of CDKs and not secondary targets. In addition to CDKs, our studies (Table S1) and previous reports (18, 62–64) have shown that ros interacts with several additional secondary kinase targets such as CK1, DYRK1A, MAPK. Therefore, we tested not only the effects of other CDK inhibitors with different specificities, but also inhibitors to secondary targets which (in principle) do not inhibit CDKs (Figure 3). Inhibition of tau phosphorylation at Thr231 and Ser202 was evident using the CDK inhibitors ros, N-&-N1 (65, 66), CR8 (64), MR4 (unpublished), SCH727965 (67), SNS-032 (68), and AT7519 (69), but not by N6-methyl-roscovitine and N6-methyl-CR8 (the kinase inactive derivatives of ros and CR8, respectively) (63) (Figure 3).

To examine whether interaction with any of the secondary kinase targets might account for the inhibition of tau phosphorylation caused by CDK inhibitors, we made use of a series of selective pharmacological inhibitors of other kinases (none of which inhibit CDKs) (Figure 3). SH-SY5Y-(P301L) cells were exposed to various concentrations of pharmacological inhibitors of CK1 [IC261 (70), DRF053 (71), D4476 (72)], DYRK1A [Leucettine 41 (73, 74), harmine (74)], CLKs [TG003 (75)] and MEK1 [UO126, PD098059 (76)]. In contrast to the potent and non-specific kinase inhibitor, staurosporine (77), none of the inhibitors to secondary kinase targets reduced tau phosphorylation, with the exception of IC261 which was active at site pSer202. Decreases in P-tau at the high (100 μM) doses of Me-(S)-CR8 and DFR053 in Figure 3 were likely the result of drug-associated cell toxicity. Overall, these results suggest that ros and other CDK inhibitors reduce tau phosphorylation at sites Thr231 and Ser202 through inhibition of CDKs rather than by interaction with secondary targets. However, the possibility that other kinases can also phosphorylate these sites cannot be excluded.

Inhibition of tau phosphorylation by ros and LiCl was tested by exposing SH-SY5Y-(P301L) cells to several concentrations of the two molecules alone or together, and measuring the level of pS202-tau and T-tau after 6 h by Western blot. Both individual treatments with ros or LiCl slightly down-regulated tau phosphorylation (Figure 4A). The slight differences between the effects of ros on S202-tau phosphorylation in Figures 2, 4 certainly arose from the fact that the 25 and 50 μM concentrations were in the descending part of the dose-response curve and therefore any slight experimental shift in concentration resulted in large differences in response. Furthermore, quantification of Western blots always remained semi-quantitative. Interestingly, although no dose-response relationship was observed with LiCl alone, the lowest and highest concentrations tested leading to the same inhibition values, tau phosphorylation was down-regulated in proportion to LiCl concentration when LiCl was combined to 30 and 50 μM ros (Figure 4B). Coefficients of drug interactions indicated a synergy between the two compounds at concentrations equal or >25 mM LiCl and 25 μM ros (Figure 4C). Ros alone exerted a moderate and dose-dependent inhibition of Ser202 phosphorylation. Surprisingly, ros seemed to be slightly antagonistic when combined to the lowest concentration of LiCl (6.25 mM). These data demonstrated that precise doses of LiCl and ros acted synergistically to reduce tau phosphorylation.

### Behavior and Cognition

The accelerating rotarod test was used to monitor motor coordination throughout the study (Figure 1) and was therefore useful for evaluating cerebellar dysfunction. Rotarod testing showed significant (*p* < 0.01) deficits in motor performance of rmCHI mice when compared to sham mice at 3 days following completion of the repetitive mild impact protocol. However, by days 7 and 10 following the last of the 4 impacts of the rmCHI protocol, the impacted mice regained full motor coordination by rotarod testing with no significant differences compared to sham and naïve mice (data not shown).

**Figure 5:**
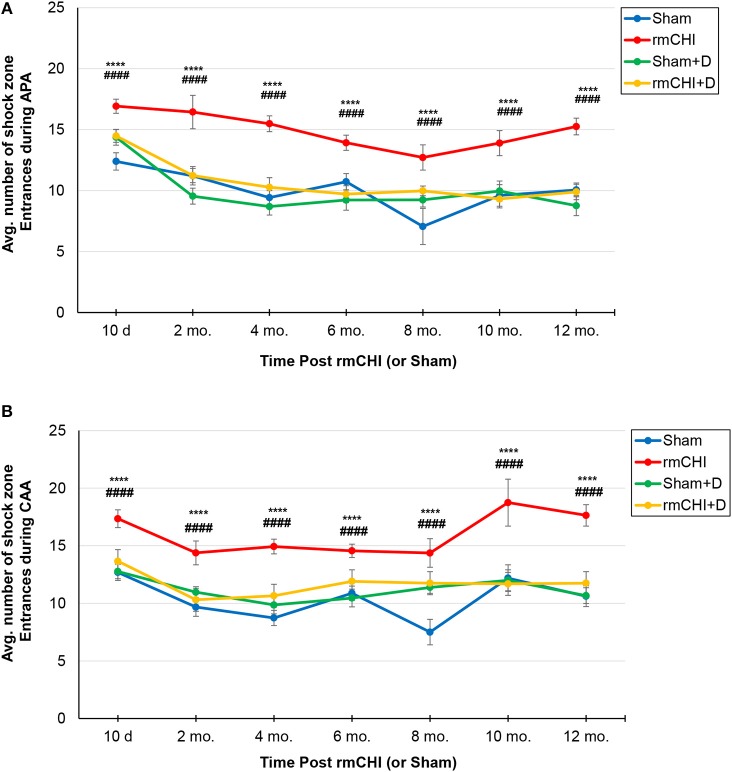
Assessment of cognitive deficits in TghTau/PS1 mice (with and without therapeutic intervention) post rmCHI (or sham). TghTau/PS1 mice were subjected to rmCHI (or sham treated) and evaluated for cognitive changes beginning at 10 days (d) following completion of the repetitive impact protocol and continuing throughout the 12 month (mo.) study. Mice were evaluated at each time point by APA **(A)** and CAA **(B)** as described in the Methods section and the average number of entrances into the shock zone were recorded. For drug studies (D), a combination of LiCl+ros was used. The data was evaluated using two-way ANOVA with Tukey's *post-hoc* test and presented as the mean ± SEM. ^*^Indicates significantly different from sham (^****^p < 0.0001). ^#^Indicates significantly different from rmCHI+drug (D) (^####^*p* < 0.0001).

Therefore, beginning at 10 days following the repetitive injury protocol (i.e., the time at which mice recovered normal motor function), and continuing throughout the study (2, 4, 6, 8, 10, and 12 months), APA and CAA testing were performed on rmCHI and sham TghTau/PS1 mice (Figure 1). At all times analyzed for APA and CAA there were no significant differences between the rmCHI vs. sham mice in the distance traveled suggesting both groups retained similar motor activity as well as a similar ability to explore the arena (data not shown). However, at most times tested in both APA and CAA, the time to first shock zone entrance in each of the four 10 min trial periods was significantly shorter and the average number of entrances per trial for APA (Figure 5A) and CAA (Figure 5B) testing formats at all times were significantly greater for rmCHI compared to sham animals. Thus, rmCHI mice exhibited impaired learning and memory by both APA and CAA since they entered the shock zone in less time and more frequently. Furthermore, compared to untreated rmCHI mice, animals that received the combination of LiCl+ros showed a highly significant reversal of both APA (Figure 5A) and CAA (Figure 5B)-tested cognitive deficits (i.e., average number of shock zone entrances) at each time point throughout the entire 12 mo. study. There were no significant differences in cognition between untreated and drug-treated sham mice throughout the entire study using either the APA (Figure 5A) or CAA (Figure 5B) formats.

**Figure 6:**
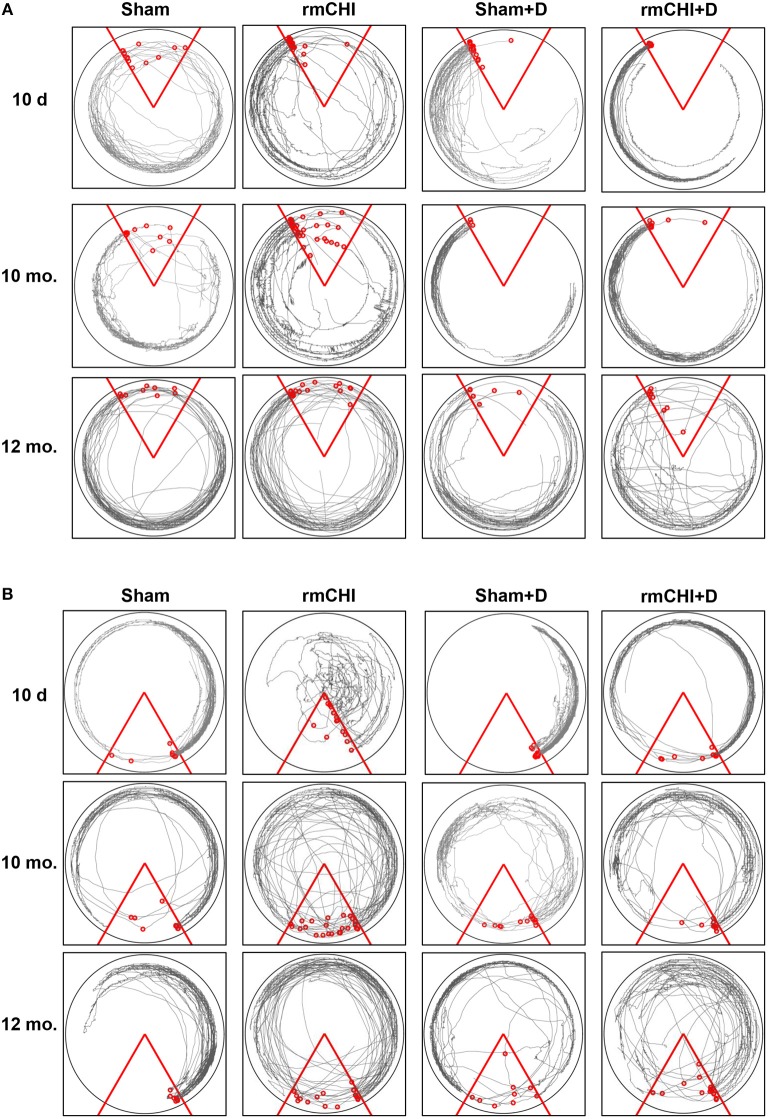
Representative tracking analysis of TghTau/PS1 mice (with and without drug treatment) post rmCHI (or sham). Movement of untreated and LiCl+ros drug-treated (D) TghTau/PS1 mice were followed and recorded for APA **(A)** and its position change of 180° for CAA **(B)** analyses protocols. Shown are representative tracking paths of individual mice during the fourth 10 min trial at the 10 day, 10, and 12 month time points post rmCHI (and sham). The testing arena is shown as a circle with the location of the triangular 60°shock zone (red lines). The continuous black lines within the arena boundary shows the actual paths taken by the mice. Within each circular arena shown, the red circles represent the location of an individual mouse from the indicated groups. The number of small red circles within each arena's triangular space represents how often the mouse entered the shock zone area and received a shock.

Tracking the actual movements taken by each mouse at each time point (10 days, 2, 4, 6, 8, 10, and 12 mos.) was analyzed as one of the APA parameters. Representative tracking of a mouse randomly selected from either the 10 day, 10, and 12 mo. groups during the fourth 10 min APA trial are shown in Figure 6A. The mouse tracking for all mice at all-time points between 10 days and 8 mos. were similar and demonstrated that, compared to sham mice, not only do the rmCHI mice display cognitive and memory deficits by entering the shock zone more frequently (as shown in Figure 5A), but also have more random, haphazard movement when tracked within the arena. Beginning at 10 mos. and extending to 12 mos., tracking of the sham mice began to show a pattern of increasingly disorganized movement approaching that of the rmCHI mice at those same time points (Figure 6A). However, interestingly, in spite of the more random movement in the tracking patterns, the sham mice continued to demonstrate shock zone avoidance at these later two time points. From 10 days to 8 mos. the rmCHI mice treated with LiCl+ros avoided the shock zone with unidirectional, organized tracking movement within the rotating arena similar to the drug-treated and untreated sham mice (not shown). At 10 mos. the drug treatment was still effective in that the rmCHI mice were still avoiding the shock zone but tracking indicated an increased random and disorganized movement. In addition, by 12 mos. the drug-treated sham mice showed signs of disorganized tracking and increased shock zone avoidance-deficit indicative of cognitive impairment. Similar to APA, rmCHI mice had a significant degree of shock zone avoidance-deficit with unfocused, disoriented tracking compared to sham mice at the 10 day time point during the CAA testing (Figure 6B). Tracking results during CAA testing format, similar to the 10 day time, were obtained at the 2–8 mo. (not shown) and 10–12 mo. (Figure 6B) times. However, the co-administration of LiCl+ros resulted in improved cognitive behavior of the mice as the rmCHI+drug (D) mice showed intentional movement to avoid entering the shock zone (Figure 6B). As with APA, CAA testing at the 12 mo. time showed both sham+D and rmCHI+D mice demonstrating more disorganized movement with less effective shock zone avoidance tracking (Figure 6B).

### Biochemical Detection of Biomarkers

Following the 4 impact (10 day) rmCHI (or sham) protocol, brain and blood were collected from the TghTau/PS1 mice throughout the 12 month study period (Figure 1). Plasma and cortical brain extracts were prepared and analyzed for T-tau and P-tau as described in Materials and Methods.

**Figure 7:**
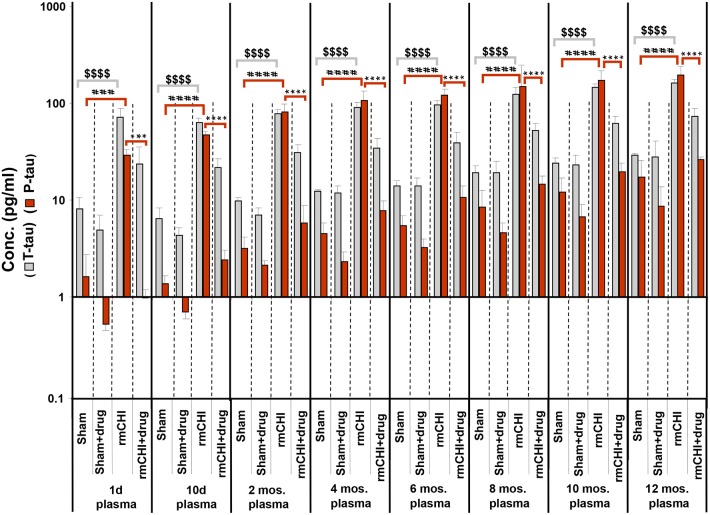
Concentrations of T-tau and P-tau in the plasma of untreated and drug (LiCl+ros) (D)-treated TghTau/PS1 mice. TghTau/PS1 mice were subjected to rmCHI (or sham-treated) and analyzed according to the study design scheme (Figure 1). Randomized groups of mice were sacrificed at each of the designated time points throughout the 12 month study and blood was collected. T-tau and P-tau biomarkers were assayed in plasma by a-EIMAF. Biomarker concentrations were determined and plotted as mean ± SEM. Analysis of statistical significance was performed by two-way ANOVA with Tukey's *post-hoc* test for: sham P-tau vs. rmCHI P-tau (^###^*p* < 0.001; ^####^*p* < 0.0001), rmCHI P-tau vs. rmCHI+drug P-tau (^***^p < 0.001; ^****^p < 0.0001), and sham T-tau vs. rmCHI T-tau (^$$$$^*p* < 0.0001).

Compared to sham mice, statistically significant (*p* < 0.001–0.0001) increases in T-tau and P-tau plasma concentrations were observed in the rmCHI mice at each time point throughout the study (Figure 7). At the 1 day time point, the rmCHI mice had significantly higher plasma concentrations of T-tau (71.2 ± 4 pg/ml) and P-tau (29.1 ± 1 pg/ml) when compared to T-tau (8.1 ± 0.7 pg/ml), and P-tau (1.6 ± 0.3 pg/ml) in the sham mice. By 12 months the plasma from rmCHI mice, compared to sham mice, was significantly greater with T-tau levels of 163 ± 3.6 pg/ml (*p* < 0.0001) and P-tau levels of 195.6 ± 10.9 (*p* < 0.0001). It is noteworthy that, compared to day 1, sham mice at 12 months also showed a significant increase in T-tau (8.10 ± 0.65 vs. 29.3 ± 0.2 pg/ml; (*p* < 0.001) and P-tau (1.6 ± 0.23 vs. 17.1 ± 2.1 pg/ml; *p* < 0.01) (Figure 7; Table 2) probably reflecting the genetically programmed neuropathology developing in these transgenic mice.

**Table 2:** Comparisons Between Untreated (−D) or Drug-Treated (+D) Sham and/or rmCHI Mice at 1 day, 6 and 12 Months.

**Group**	**Plasma**		**Cortex**	
	**Change**	***p*-value**	**Change**	***p*-value**
**T-tau**				
1 d sham (−D) vs. 6 mos. sham (-D)	ns	–	inc.	<0.05
1 d sham (−D) vs. 6 mos. rmCHI (-D)	inc.	<0.0001	inc.	<0.0001
1 d sham (−D) vs. 12 mos. sham (-D)	inc.	<0.001	inc.	<0.0001
1 d sham (−D) vs. 12 mos. rmCHI (-D)	inc.	<0.0001	inc.	<0.0001
1 d rmCHI (+D) vs. 12 mos. rmCHI (+D)	dec.	<0.0001	inc.	<0.0001
1 d rmCHI (−D) vs. 6 mos. rmCHI (-D)	inc.	<0.001	inc.	<0.0001
6 mos. sham (−D) vs. 12 mos. sham (-D)	ns	–	ns	–
6 mos. sham (−D) vs. 12 mos. rmCHI (-D)	inc.	<0.0001	inc.	<0.0001
6 mos. rmCHI (−D) vs. 12 mos. rmCHI (-D)	inc.	<0.0001	inc.	<0.0001
6 mos. rmCHI (+D) vs. 12 mos. rmCHI (+D)	inc.	<0.0001	ns	–
**P-tau**				
1 d sham (−D) vs. 6 mos. sham (-D)	ns	–	ns	–
1 d sham (−D) vs. 6 mos. rmCHI (-D)	inc.	<0.0001	inc.	<0.0001
1 d sham (−D) vs. 12 mos. sham (-D)	inc.	<0.01	inc.	<0.01
1 d sham (−D) vs. 12 mos. rmCHI (-D)	inc.	<0.0001	inc.	<0.01
1 d rmCHI (+D) vs. 12 mos. rmCHI (+D)	inc.	<0.01	inc.	<0.001
1 d rmCHI (−D) vs. 6 mos. rmCHI (-D)	inc.	<0.0001	inc.	<0.0001
6 mos. sham (−D) vs. 12 mos. sham (-D)	ns	–	ns	–
6 mos. sham (−D) vs. 12 mos. rmCHI (-D)	inc.	<0.0001	inc.	<0.0001
6 mos. rmCHI (−D) vs. 12 mos. rmCHI (-D)	inc.	<0.0001	inc.	<0.01
6 mos. rmCHI (+D) vs. 12 mos. rmCHI (+D)	ns	–	ns	–

*ns, not statistically significant*.

*all p ≤ 0.05 indicates statistically significant change*.

Notable are the patterns of increasing T-tau and P-tau plasma levels post rmCHI over the 12 mo. study expressed as the P-tau:T-tau ratios (Table 3). By 2 mos. post injury the P-tau:T-tau ratio approached 1. Whereas, at the 1 day and 10 day time points, the P-tau:T-tau ratio was <1 and from 4 to 12 months the ratio was >1 indicating a dynamic reversal in the rates of T-tau and P-tau concentrations. From 4 to 12 mos. the P-tau:T-tau ratios were fairly constant indicating that the rates of both P-tau and T-tau concentrations were increasing similarly.

**Table 3:** P-tau:T-tau^a^.

**Time Point^b^**	**Sham**	**Sham ± Drug**	**rmCHI**	**rmCHI ± Drug**
	**Cortex; Plasma**	**Cortex; Plasma**	**Cortex; Plasma**	**Cortex; Plasma**
1 d	0.42; 0.20	0.38; 0.10	0.81; 0.41	0.59; 0.04
10 d	0.46; 0.22	0.37; 0.16	0.68; 0.73	0.15; 0.11
2 mos.	0.41; 0.33	0.46; 0.30	0.62; 1.06	0.12; 0.19
4 mos.	0.38; 0.37	0.40; 0.20	0.47; 1.18	0.12; 0.22
6 mos.	0.38; 0.39	0.31; 0.23	0.30; 1.25	0.15; 0.28
8 mos.	0.36; 0.44	0.26; 0.24	0.31; 1.22	0.11; 0.28
10 mos.	0.40; 0.51	0.28; 0.29	0.30; 1.18	0.13; 0.33
12 mos.	0.40; 0.58	0.26; 0.30	0.26; 1.20	0.12; 0.36

a *Ratios are calculated based on mean ± SEM values*.

b *Time following last of four rmCHI impacts*.

The LiCl+ros treated TghTau/PS1 mice subjected to rmCHI showed statistically significant reductions in the plasma concentrations of P-tau at the corresponding time points (Figure 7) beginning at day 1 with 23.8 ± 2.9 and 0.98 ± 0.38 for T-tau and P-tau, respectively, and extending to 12 months (T-tau: 73.3 ± 3.7 pg/ml; P-tau 26.4 ± 0.34 pg/ml (Figure 7; Table 2). Furthermore, the drug treatment prevented the dynamic changes in the P-tau and T-tau concentrations seen in the untreated rmCHI mice resulting in P-tau:T-tau ratios of < 1 at all-time points (Table 3).

**Figure 8:**
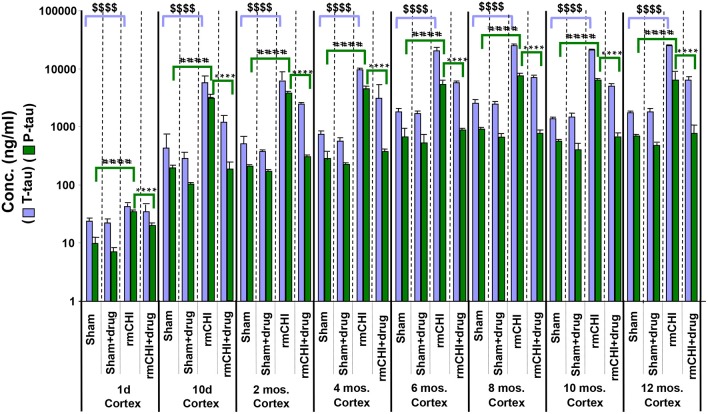
Concentrations of T-tau and P-tau in the cortical brain regions of untreated and drug (LiCl+ros) (D)-treated TghTau/PS1 mice. TghTau/PS1 mice were subjected to rmCHI (or sham-treated) and analyzed as summarized in the study design (Figure 1). Randomized groups of mice were sacrificed at each of the designated time points throughout the 12 month study and the brain cortex was dissected and frozen. Extracts of the cortical brain regions were prepared followed by T-tau and P-tau quantitation by EIMAF. Biomarker concentrations were determined and data plotted as mean ± SEM. Analysis of statistical significance was performed by two-way ANOVA with Tukey's *post-hoc* test for: sham P-tau vs. rmCHI P-tau (^####^*p* < 0.0001), rmCHI P-tau vs. rmCHI+drug P-tau (^****^p < 0.0001), and sham T-tau vs. rmCHI T-tau (^$$$$^*p* < 0.0001).

The absolute values and the rates at which the levels of T-tau and P-tau increased following rmCHI differed in the cortex (Figure 8) compared to the plasma (Figure 7). While the increases in P-tau were greater than T-tau in plasma, the opposite was observed in the cortex as exemplified by the lower P-tau:T-tau ratios for cortex in rmCHI mice (Table 3).

Similar to our findings in the plasma, the increased levels of cortical brain T-tau and P-tau at each time point in the rmCHI groups were reversed by drug treatment at statistically significant levels (Figure 8). The extent of the reversal was more pronounced for P-tau than T-tau and approaching the P-tau levels in the drug-treated sham mice (Figure 8).

Over the course of the study, the changes in the T-tau and P-tau patterns were, in most cases, similar. As an example, the altered biomarker patterns and significance of the changing T-tau and P-tau levels in the plasma and cortex were compared between day 1, 6, and 12 mos. (Table 2). This suggests that biomarker changes in the blood might be a useful barometer to ascertain abnormal protein processing in the brain. Interestingly, the P-tau levels in the drug-treated mice at 6 and 12 mos were not statistically different suggesting a steady state generation and/or degradation of P-tau in these aging mice.

For all behavior, cognition and biomarker studies of untreated and drug-treated mice throughout the 12 mo. study period, we did not find any gender-associated significant differences both within and/or between rmCHI and sham groups.

## Discussion

The ability to reliably diagnose TBI is directly related to the severity of the head trauma. Moderate and severe TBI where there are intracranial bleedings or brain lesions can be diagnosed by clinical exams and neuroimaging. However, in mTBI where there is absence of gross pathological brain lesions, brain hemorrhage and abnormal neuroimaging, the use of biomarkers is valuable. Protein biomarkers in biofluids would be useful not only for determining TBI severity but also to aid in identifying the source (neurons, axons, astrocytes) of the pathophysiology. Although CSF is the most desired biofluid since it directly interfaces with the brain and closely reflects brain alterations, blood is more accessible but with biomarker levels more difficult to detect. Detection of blood-based TBI biomarkers are complicated by the involvement of the BBB. The transport of brain molecules into the peripheral circulation depends on the solubility, molecular weight, and size of proteins. Depending on the severity and type of injury, the BBB becomes more permeable, allowing cellular degradation products and other molecules to enter more freely into the systemic circulation. An intact BBB, which restricts CNS protein diffusion into blood, combined with dilution of low levels of brain-specific proteins in the blood and extracellular fluid, negatively affects reliable and consistent detectability (78). This is less of an issue with sTBI especially during the acute phase, but a major factor, which requires ultra-sensitive detection technology, with mTBI where the head trauma may only marginally compromise the BBB. In addition, CNS-derived proteins might be degraded by blood-containing proteases causing changes in their biochemical and immunological properties reducing their detectability (79).

The temporal pattern (emergence, persistence and decline) of biomarker concentrations are valuable for confirming the appearance and extent of a brain injury as well as identifying a timeline for patients to return to normal activity. GFAP is almost exclusively expressed in astrocytes (80) and serves as a biomarker for astroglial injury. Elevated GFAP levels has been reported to be a diagnostic and prognostic biomarker for acute TBI. In modTBI and sTBI, GFAP levels were elevated in CSF and serum, particularly in patients who experienced an unfavorable outcome (81). GFAP levels in CNS have been suggested to improve TBI outcome prediction models and may serve as a marker of intracranial injury (82, 83). GFAP in CSF has been reported to have higher diagnostic accuracy than S100β (84) and was shown to accurately differentiate mTBI patients from controls (85). GFAP concentrations in serum during acute TBI has been used to discriminate between mTBI and sTBI (86–88).

Plasma levels of GFAP was able to discriminate TBI patients from controls in a study where the majority of patients had mTBI although up to one quarter of TBI patients had GFAP levels below the lower limit of detection (31). UCH-L1 is a brain-specific biomarker whose levels correlate with injury severity (89, 90) but has shown inconsistent results with mTBI (29). The concentration of UCH-L1 increases and decreases within a few hours after injury, with GFAP detection lasting further in the acute phase (91). Increased sensitivity and specificity for diagnosing acute TBI were obtained when GFAP was combined with UCH-L1, thus supporting the use of a combination of biomarkers for diagnosis and prognosis during acute TBI (31). In a study where 584 trauma patients were examined within 4 h of injury, GFAP and/or UCH-L1 were both significantly higher in CT negative mTBI patients than in patients with orthopedic injuries (86, 92). In contrast, using a different assay Posti et al. (32) reported that no significant differences in the blood concentrations of GFAP or UCH-L1 between mTBI patients with a negative CT and orthopedic control patients.

In a study where serum and plasma levels of S100β, neuron-specific enolase (NSE), and T-tau were monitored in ice hockey players prior to and after concussions (93), time-dependent changes in plasma T-tau and serum S100β levels (biomarkers for axonal and astroglial injury, respectively), but not NSE, correlated best with concussion. The return of T-tau and S100β to pre-injury levels correlated well with successful rehabilitation and timing of return to play. Sensitive methods, such as Simoa (Quanterix, Lexington, MA), claim single-molecule detection (SMD) and have been used to measure serum and plasma T-tau concentrations (94). Using Simoa, the levels of plasma T-tau in hockey players following games was found to be greater than baseline levels prior to hockey games (95). Another study using Simoa reported long-term increases in plasma T-tau in military personnel who had multiple TBIs and chronic post-concussive disorder suggesting an association between T-tau and neurological symptoms (96). Further, using Simoa to compare plasma T-tau levels with controls in symptomatic former National Football League players, increased repetitive head injury exposure predicted higher later-life plasma T-tau concentrations (97).

However, the usefulness of T-tau as a tauopathy biomarker for TBI-associated neuronal damage is still unclear. It has been reported (98) that in human brain samples from patients with either sTBI or modTBI, the levels of P-tau, and not T-tau, correlated with TBI severity and the extent of cognitive deficits as measured by the Glasgow Coma Scale (GCS). P-tau pathology throughout the human brain has been observed in late-stage CTE, which is considered a consequence of rmTBIs (99). Pathological tau deposition in the brain is not uncommon among neurodegenerative diseases including AD. Using a different ultra-sensitive assay technology, P-tau levels have been shown to be elevated in serum longer than T-tau concentrations (7). Further, plasma P-tau levels and P-tau/T-tau ratio during the acute phase and chronic TBI were superior to T-tau levels for discriminating the severity and status of neurotrauma patients from healthy controls (36). While P-tau may not be specific for mTBI, a recent study of 196 human patients with acute TBI found that in single time point blood samples the ratio of P-tau to T-tau in plasma served as a good diagnostic and prognostic marker for acute TBI across different severities, as measured by GCS scores. Further, in the same study, 21 patients with severe TBI and receiving inpatient rehabilitation, P-tau levels and P-tau:T-tau ratios show more robust and sustained elevations among patients with chronic TBI compared with T-tau levels alone (36). This suggests that tau has the potential as a biomarker of mTBI and possibly for CTE. In addition, a mouse model subjected to rmCHI exhibited abnormal behavior, cognitive deficits and pathological changes including astrogliosis, microglial activation and P-tau immunoreactivity which persisted and increased throughout a 6 month study period (5, 6). The distribution of P-tau pathology described in the studies was similar to those reported in CTE cases (100, 101).

Numerous rodent models have been developed in attempts to replicate the features of CTE such as how the concussions are induced, the injury severity, the number of head impacts and the time between repetitive injuries [reviewed by McAteer et al. (102)]. As a result, this has led to difficulties in the ability to replicate critical aspects of CTE such as: increased P-tau, CNS progression of tau and NFT pathology, and development of behavioral and cognitive deficits. Our studies included brain and blood sample collection and analyses over a 12 month period. Previous reports using rat and mouse models of TBI have demonstrated that blood concentrations of biomarkers are associated with a dynamic continuum of physiological and pathological changes (24, 103). This highlights the importance of serial sampling of biofluids and avoids any misinterpretation that may arise of single post-injury sampling since this may not capture dynamically changing concentrations of biofluid biomarkers. Therefore, the absence of serial sampling can lead to an oversight of pathophysiological processes, misleading of disease progression, and failure to realize where therapy could be most effective.

Our accomplished goals were to develop an experimental TBI model of rmCHI where T-tau and P-tau concentrations can be monitored in blood over time, to determine whether our findings in blood reflect the qualitative and/or quantitative biomarker changes in the CNS, and assess whether they correlate with cognitive changes during early and chronic stages. It is interesting that although the T-tau and P-tau concentrations increased in both brain and blood as a result of rmCHI, the dynamics associated with these increases were not the same possibly reflecting selectivity of the BBB. We then investigated the effectiveness of our therapeutic intervention regimen of administering ros and LiCl to target and reduce tau phosphorylation. This treatment was effective at reversing T-tau and P-tau phosphorylation in brain and blood over a 12 month period and correlated with diminished cognitive deficits. Previous studies using rat and mouse models of TBI have reported ros treatment post TBI reduces cognitive deficits by decreasing both lesion volume and microglia activation thereby inhibiting neurodegeneration and neuroinflammation (104). The beneficial effects of lithium in TBI models have been reported by several groups (20, 46, 105–110). Ros and lithium inhibit protein kinases with non-overlapping selectivity: ros targets mainly CDKs and CK1s, while lithium inhibits GSK-3. These kinases phosphorylate tau at multiple and different sites [review in Kimura et al. (111)].

Our studies reported the effects of simultaneous inhibition of the two main protein kinases responsible for tau hyperphosphorylation, GSK-3β and cdk5. Several additional points should be noted from our findings: (i) Our data lends support to the view that the physiological functions of these protein kinases on tau extend beyond that of phosphorylation since the use of LiCl+ros not only reduced the levels of P-tau in brain and blood but also, to a lesser degree, also decreased the concentrations of T-tau (Table 4), (ii) Our studies also demonstrated the therapeutic efficacy of the LiCl+ros co-treatment not only at the early stages, but also during the chronic stages following rmCHI (Table 4), (iii) Although the TghTau/PS1 mice are genetically programmed to develop a tauopathy, the increase in P-tau levels in the sham mice never approached the levels in the rmCHI mice indicating that head trauma accelerates both early and long-term neuropathological processes. Furthermore, treatment of the sham mice with LiCl+ros did, in fact, decrease the P-tau levels which was especially noticeable at the later time points (Table 4) where the natural tauopathy in these transgenic mice would be more evident.

**Table 4:** Percent decrease of P-tau and T-tau in drug-treated compared to untreated mice.

	**Sham**				**rmCHI**			
	**Plasma**		**Cortex**		**Plasma**		**Cortex**	
	**P-tau**	**T-tau**	**P-tau**	**T-tau**	**P-tau**	**T-tau**	**P-tau**	**T-tau**
1 d	29	40	25	8	97	66	43	21
10 d	20	33	48	14	95	66	94	79
2 mos.	34	29	18	15	93	60	92	60
4 mos.	49	8	19	12	93	62	92	68
6 mos.	42	0	23	8	91	60	86	72
8 mos.	44	0	30	1	90	57	90	77
10 mos.	42	0	29	0	89	58	89	76
12 mos.	47	3	31	0	87	55	88	75

Epilepsy has been hypothesized as a tauopathy (112) due to increased levels of P-tau (113, 114) which has been reported to be the result of increased GSK-3β and cdk5 activities (115). Further, in both temporal lobe epilepsy patients and an epileptic rat model, the epilepsy-induced axonal impairment is characterized by increased P-tau and decreased T-tau (115). Inhibiting GSK-3β or cdk5 in the epileptic rats using LiCl or ros, respectively, reversed axonal impairment. GSK-3β inhibition lowered the relative P-tau levels by reversing the decrease of T-tau while cdk5 inhibition lowered P-tau levels without affecting T-tau (115). This suggests that in our rmCHI studies, by combining LiCl and ros it is possible that we exacerbated and/or prolonged the P-tau inhibition by simultaneously interfering with different phosphorylation mechanisms or pathways. We found some evidence for additivity (moderate doses) and synergy (highest doses) of LiCl and ros *in vitro* (Figure 4), but also some slight antagonism at the lowest doses of both ros and LiCl. Although it is not possible to extrapolate *in vivo* results from *in vitro* results, especially in terms of dosing, our *in vivo* results are suggestive for at least some additivity, and possibly some synergy, between the two co-treatments, especially as the targets of both drugs do not overlap. *In vitro*, additivity and synergy were observed at doses at which tau phosphorylation was inhibited *in vivo*. It would be of interest to investigate the cognitive and tau phosphorylation effects of LiCl and ros individually *in vivo* and to compare their effects with the combined treatment in our rmCHI model.

Currently CTE can only be diagnosed neuropathologically through the post-mortem detection of P-tau containing NFTs. Therefore, the development of P-tau in animals following injury is a basic requirement for any acceptable experimental model. In addition, an experimental model can serve to address factors associated with disease development which includes: role of impact severity, time interval between impacts, age at which impacts occur, and the total number of impacts required. Other important variables such as the brain region of impacts, and effect of genetics also need to be addressed. An experimental model of CTE must display neurotrauma-induced behavioral changes and exhibit cognitive deficits which persist or worsen at chronic time points post-injury.

Validating the use of transgenic mice to study rmCHI is necessary to support translational relevance. Luo et al. (116) developed and characterized rmCHI in wild-type C57BL/6J mice and observed a cumulative increase in astrocytosis from rmCHI. The rmCHI mice showed a significant impairment in spatial learning and memory when tested at 2 and 6 months after injury and increased P-tau immunoreactivity in post-mortem pathological examinations. These findings were consistent with the deficits and pathology associated with mTBI in humans.

We described a non-mutated human tau-expressing mouse line which is being used for the first time to study the neuropathophysiology associated with rmCHI throughout the early and chronic time periods. We described the utility of early and chronic brain and blood-derived biochemical biomarker analysis and the interesting dynamics associated with changes in tau. We further describe a drug treatment regimen and demonstrate its effectiveness by correlating levels of biochemical biomarkers in the cortical brain regions and plasma with changes in cognitive deficits. It should be realized that our findings using the cortical brain regions may not entirely reflect the neuropathophysiological processes in other brain regions following head trauma. Biochemical biomarker studies on additional brain regions are necessary to assess qualitative and quantitative similarities and/or differences with the cortex. In addition, neuropathological studies utilizing immunohistochemistry would aid in identifying the brain regions of biomarker localization in untreated and drug-treated mice subjected to rmCHI. We anticipate that our studies will provide a foundation for establishing an *in vivo* model of CTE and other TBI-associated tauopathies. In addition, our results support the exploration of CDKs and GSK-3 drug combinations as potential therapeutic treatments for CTE and other TBI-associated tauopathies.

## Data Availability

The data supporting the conclusions of this manuscript will be made available by the authors, without undue reservation, to qualified researchers upon request.

## Ethics Statement

This study was carried out in accordance with the recommendations of the National Institutes of Health guide for the care and use of laboratory animals, approval from the Department of Defense Animal Care and Use Review Office and under the protocol approval, supervision, and compliance with the SUNY Downstate Institution Animal Care and Use Committee. Animal studies were carried out following the ARRIVE guidelines for the design, analysis, and reporting of scientific research.

## Author Contributions

RR and KW conceived and designed the experiments. TW and LM provided resources and guidance on experimental protocols. AC provided animal husbandry and mouse genotyping. NO synthesized roscovitine. RR, DS, BC, MC, LV, ML, and LM performed the biological experiments and contributed to the analysis, and interpretation of data. RR, DS, and LM prepared figures and wrote the manuscript. All authors discussed, commented on, and approved the manuscript.

### Conflict of Interest Statement

LM is an inventor on two patents covering roscovitine, and heading a biotech company which develops roscovitine for the treatment of cystic fibrosis. The remaining authors declare that the research was conducted in the absence of any commercial or financial relationships that could be construed as a potential conflict of interest.
