# Supplementary material for: Novel Mouse Tauopathy Model for Repetitive Mild Traumatic Brain Injury: Evaluation of Long-Term Effects on Cognition and Biomarker Levels After Therapeutic Inhibition of Tau Phosphorylation
Source: Front Neurol. 2019 Mar 11;10:124. doi: 10.3389/fneur.2019.00124 (PMC6421297; doi:10.3389/fneur.2019.00124)
Supplement: Supplementary file 1 [file Table_1.pdf]

Supplementary Data

Table S1. Kinases enriched from mouse brain using the KinAffinity® matrix.

Uniprot ID	Protein	Synonym
Q3UHJ0	Adaptor-associated kinase 1	AAK1
P00520	Abelson murine leukemia viral oncogene homolog 1	ABL
P37172	Activin receptor type I	ACVR1
Q62407	Aortic preferentially expressed protein 1	APEG1
Q4JIM5	Abelson murine leukemia viral oncogene homolog 2	ARG
Q91Z96	BMP-2-inducible protein kinase	BIKE
O35607	Bone morphogenetic protein receptor type II	BMPR2
Q5RJI5	BR serine/threonine-protein kinase 1	BRSK1
Q69Z98	BR serine/threonine-protein kinase 2	BRSK2
Q8BW96	Calcium/calmodulin-dependent protein kinase type 1D	CAMK1D
P11798	Calcium/calmodulin-dependent protein kinase type II subunit alpha	CAMK2A
P28652	Calcium/calmodulin-dependent protein kinase type II subunit beta	CAMK2B
Q6PHZ2	Calcium/calmodulin-dependent protein kinase type II subunit delta	CAMK2D
Q923T9	Calcium/calmodulin-dependent protein kinase type II subunit gamma	CAMK2G
P08414	Calcium/calmodulin-dependent protein kinase type IV	CAMK4
Q8VBY2	Calcium/calmodulin-dependent protein kinase kinase 1	CAMKK
Q8C078	Calcium/calmodulin-dependent protein kinase kinase 2	CAMKK2
Q3UU96	CDC42-binding protein kinase alpha	CDC42BPA
Q7TT50	CDC42-binding protein kinase beta	CDC42BPB
Q80YP0	Cell division protein kinase 3	CDK3
P30285	Cell division protein kinase 4	CDK4
P49615	Cell division protein kinase 5	CDK5
Q99J95	Cell division protein kinase 9	CDK9
Q69ZA1	Cell division protein kinase 13<br>CDC2-related protein kinase 5	CDK13
O35495	Cell division protein kinase 14	CDK14
Q04735	Cell division protein kinase 16	CDK16
Q8K0D0	Cell division protein kinase 17	CDK17
Q04899	Cell division protein kinase 18	CDK18
Q60680	Conserved helix-loop-helix ubiquitous kinase	CHUK
P41241	C-Src kinase	CSK
Q8BK63	Casein kinase I isoform alpha	CSNK1A1
Q9DC28	Casein kinase I isoform delta	CSNK1D
Q9JMK2	Casein kinase I isoform epsilon	CSNK1E
Q8BTH8	Casein kinase I isoform gamma-1	CSNK1G1
Q8BVP5	Casein kinase I isoform gamma-2	CSNK1G2
Q8C4X2	Casein kinase I isoform gamma-3	CSNK1G3
Q60737	Casein kinase II subunit alpha 1	CSNK2A1
O54833	Casein kinase II subunit alpha 2	CSNK2A2
Q9JLM8	Doublecortin-like and CAM kinase-like 1	DCAMKL1
Q03146	CD167 antigen-like family member A (Cak)	DDR1
Q61214	Dual specificity tyrosine-phosphorylation-regulated kinase 1A	DYRK1A
Q9Z188	Dual specificity tyrosine-phosphorylation-regulated kinase 1B	DYRK1B
Q01279	Epidermal growth factor receptor	EGFR
Q03137	Ephrin type-A receptor 4	EPHA4
Q60629	Brain-specific kinase (Bsk)	EPHA5
Q62413	EPH homology kinase 2 (Ehk2)	EPHA6
Q8CBF3	Ephrin type-B receptor 1	EPHB1
P54763	Ephrin type-B receptor 2	EPHB2
O08644	Ephrin type-B receptor 6 (Cekl)	EPHB6
Q63844	Extracellular signal-regulated kinase 1	ERK1
P63085	Extracellular signal-regulated kinase 2	ERK2
Q80Y86	Extracellular signal-regulated kinase 7	ERK7
P34152	Focal adhesion kinase 1	FAK
P70451	p94-Fer	FER

P16092	Basic fibroblast growth factor receptor 1	FGFR1
P21803	Fibroblast growth factor receptor 2 (Bek)	FGFR2
Q61851	Fibroblast growth factor receptor 3	FGFR3
P09581	Macrophage colony-stimulating factor 1 receptor (Csf1r)	FMS
P39688	p59-Fyn	FYN
Q99KY4	Cyclin-G-associated kinase	GAK
Q2NL51	Glycogen synthase kinase-3 alpha	GSK3A
Q9WV60	Glycogen synthase kinase-3 beta	GSK3B
O88351	I-kappa-B kinase 2 (Ikbkb)	IKKB
O55222	Integrin-linked protein kinase	ILK
P52332	Janus kinase 1	JAK1
Q91Y86	c-Jun N-terminal kinase 1	JNK1
Q9WTU6	c-Jun N-terminal kinase 2	JNK2
Q61831	c-Jun N-terminal kinase 3	JNK3
P05532	Mast/stem cell growth factor receptor	KIT
Q61097	Kinase suppressor of Ras 1	KSR
Q3UVC0	Kinase suppressor of Ras 2	KSR2
P53668	KIZ-1	LIMK
Q9WTK7	Serine/threonine-protein kinase 11	LKB1
Q5S006	Leucine-rich repeat serine/threonine-protein kinase 2	LRRK2
P25911	Tyrosine-protein kinase Lyn	LYN
P31938	Dual specificity mitogen-activated protein kinase kinase 1	MAP2K1
Q63932	Dual specificity mitogen-activated protein kinase kinase 2	MAP2K2
P47809	C-JUN N-terminal kinase kinase 1	MAP2K4
Q9WVS7	Dual specificity mitogen-activated protein kinase kinase 5	MAP2K5
P70236	Dual specificity mitogen-activated protein kinase kinase 6	MAP2K6
Q66L42	Mitogen-activated protein kinase kinase kinase 10	MAP3K10
Q60700	Dual leucine zipper bearing kinase	MAP3K12
Q1HKZ5	Mitogen-activated protein kinase kinase kinase 13	MAP3K13
O35099	Apoptosis signal-regulating kinase 1 (Ask1)	MAP3K5
Q3U1V8	Mitogen-activated protein kinase kinase kinase 9	MAP3K9
Q99JP0	Germinal center kinase-related protein kinase	MAP4K3
P97820	HPK/GCK-like kinase HGK	MAP4K4
Q9JM52	GCK family kinase MiNK	MAP4K6
Q9WUI1	Mitogen-activated protein kinase 11	MAPK11
P47811	Mitogen-activated protein kinase 14	MAPK14
Q8VHJ5	ELKL motif serine/threonine-protein kinase 3 (Emk3)	MARK1
Q05512	ELKL motif kinase 1 (Emk)	MARK2
Q03141	ELKL motif kinase 2 (Emk2)	MARK3
Q8CIP4	MAP/microtubule affinity-regulating kinase 4 (Kiaa1860)	MARK4
Q9R1L5	Microtubule-associated serine/threonine-protein kinase 1 (Kiaa0973)	MAST1
Q3U214	Microtubule-associated serine/threonine-protein kinase 3 (Kiaa0561)	MAST3
Q9ESL4	Leucine zipper- and sterile alpha motif kinase ZAK	MLTK
O88697	Myristoylated and palmitoylated serine/threonine-protein kinase (Edpk)	MPSK1
Q8C050	90 kDa ribosomal protein S6 kinase 5	MSK1
Q9Z2B9	90 kDa ribosomal protein S6 kinase 4	MSK2
Q9JI11	Mammalian STE20-like protein kinase 1	MST1
Q9JLN9	FK506-binding protein 12-rapamycin complex-associated protein 1 (Frap)	MTOR
Q8VCR8	Myosin light chain kinase 2, skeletal/cardiac muscle	MYLK2
Q8K1R7	Nercc1 kinase	NEK9
Q641K5	NUAK family SNF1-like kinase 1 (Kiaa0537)	NUAK1
P18653	90 kDa ribosomal protein S6 kinase 1	P90RSK1
Q9WUT3	90 kDa ribosomal protein S6 kinase 2	P90RSK2
P18654	90 kDa ribosomal protein S6 kinase 3	P90RSK3
Q8BTW9	p21-activated kinase 4 (Kiaa1142)	PAK4
Q8C015	p21-activated kinase 5	PAK5
Q9Z2A0	3-phosphoinositide-dependent protein kinase 1	PDK1
P07934	Phosphorylase b kinase gamma catalytic chain, skeletal muscle isoform	PHKG
Q9DB30	Phosphorylase b kinase gamma catalytic chain	PHKG2
P05132	cAMP-dependent protein kinase catalytic subunit alpha	PKACA
P68181	cAMP-dependent protein kinase catalytic subunit beta	PKACB
P20444	Protein kinase C alpha type	PKCA

P68404	Protein kinase C beta type	PKCB
P63318	Protein kinase C gamma type	PKCC
P28867	nPKC-delta	PKCD
P16054	nPKC-epsilon	PKCE
Q62101	nPKC-D1	PKCM
Q02111	nPKC-theta	PKCQ
P70268	Protein kinase C-like 1	PKN
Q5EG47	5-AMP-activated protein kinase catalytic subunit alpha-1	PRKAA1
Q8BRK8	5-AMP-activated protein kinase catalytic subunit alpha-2	PRKAA2
Q8BZ03	nPKC-D2	PRKD2
P0C605	cGMP-dependent protein kinase 1	PRKG1
Q61410	cGMP-dependent protein kinase 2	PRKG2
Q9QVP9	Proline-rich tyrosine kinase 2	PYK2
Q6P4S6	Salt-inducible kinase 3 (Kiaa0999)	QSK
P58801	Receptor-interacting serine/threonine-protein kinase 2	RIPK2
P70336	p164 ROCK-2 (Rock2)	ROCK2
Q9QZS5	Serine/threonine-protein kinase Sgk2	SGK2
O54988	Etk4 (Kiaa0204)	SLK
Q8VDU5	SNF1-related kinase	SNRK
P05480	Neuronal proto-oncogene tyrosine-protein kinase Src	SRC
O54781	Serine/arginine-rich protein-specific kinase 2	SRPK2
Q5F2E8	Serine/threonine-protein kinase TAO1 (Kiaa1361)	TAOK1
Q6ZQ29	Serine/threonine-protein kinase TAO2 (Kiaa0881)	TAOK2
Q9WUN2	Serine/threonine-protein kinase TBK1	TBK1
O55047	Serine/threonine-protein kinase tousled-like 2	TLK2
P83510	Traf2 and NCK-interacting protein kinase (Kiaa0551)	TNIK
Q0KL02	Triple functional domain protein	TRIO
P15209	BDNF/NT-3 growth factors receptor (Ntrk2)	TRKB
Q6VNS1	GP145-TrkC (Ntrk3)	TRKC
A2ASS6	Connectin	TTN
Q9QY01	Serine/threonine-protein kinase ULK2 (Kiaa0623)	ULK2
Q3U3Q1	Serine/threonine-protein kinase ULK3	ULK3
Q3UH66	Protein kinase lysine-deficient 2 (Kiaa1760)	WNK2
Q04736	p61-Yes	YES
Q6PF93	Phosphatidylinositol 3-kinase catalytic subunit type 3	PIK3C3
Q9Z1T6	1-phosphatidylinositol-3-phosphate 5-kinase	PIKFYVE
O70172	1-phosphatidylinositol-5-phosphate 4-kinase 2-alpha	PIP4K2A
Q80XI4	1-phosphatidylinositol-5-phosphate 4-kinase 2-beta	PIP4K2B
Q91XU3	Phosphatidylinositol-5-phosphate 4-kinase type II gamma	PIP4K2C
P70181	Phosphatidylinositol-4-phosphate 5-kinase type I alpha	PIP5K1A
O70161	Phosphatidylinositol-4-phosphate 5-kinase type I gamma	PIP5K1C
